# Utility of Xpert Carba-R® in diagnostic laboratory for early detection of carbapenems resistant Enterobacteriaceae

**DOI:** 10.1186/2047-2994-4-S1-P117

**Published:** 2015-06-16

**Authors:** S Aljohani, T Al Enizi

**Affiliations:** 1 College of Science and Health Professions, King Saud Bin Abdulaziz University for Health Sciences, Riyadh, Saudi Arabia; 2 College of Medicine, KSAU-HS, Saudi Arabia

## Introduction

The emergence and global spread of carbapenem-resistant Enterobacteriaceae (CRE) is of great concern to health services worldwide. These bacteria are often resistant to all beta-lactam antibiotics and frequently co-resistant to most other antibiotics, leaving very few treatment options. Healthcare facilities need the ability to test high-risk patients and get an accurate result quickly during or prior to the admission process for better patient and bed management. Traditional enriched culture methods are laborious, taking up to 72 hours for a result.

## Objectives

In our study, we evaluated the Carba-R kit from Cepheid GeneXpert, which is a rapid, and a comprehensive test that detects and differentiates the most prevalent carbapenemases (KPC, NDM, VIM, OXA-48 and IMP-1) is important for a successful infection control program.

## Methods

We tested 60 resistant isolates as per manufacturer’s instructions. We used either known controls or other samples in which has been tested by Research Unit for genotyping detections.

All tests have been performed as per manufacturer instructions.

## Results

Of the 60 samples, all samples were for *Klebsiella pnemoniae*. 16 were negative as expected and 44 were positive as follow: TEN for NDM (22.7%), TWO for VIM (0.045%) and THE REST for OXA48 (72.7%).

The assay correctly identified 56 of the samples and failed to identify FOUR isolates, which has multiple genetic resistant mechanisms.

## Conclusion

Xpert Carba-R is a very promising tool for rapid screening of CRE, assay at small scoop failed to detect multiple resistant mechanism, or mechanism at which CRE due to other rare genotypes not listed in the panel. Further studies needed to elaborate more on this assay.

## Disclosure of interest

None declared.

